# Deontological Dilemma Response Tendencies and Sensorimotor Representations of Harm to Others

**DOI:** 10.3389/fnint.2017.00034

**Published:** 2017-12-12

**Authors:** Leonardo Christov-Moore, Paul Conway, Marco Iacoboni

**Affiliations:** ^1^Ahmanson-Lovelace Brain Mapping Center, Brain Research Institute, University of California, Los Angeles, Los Angeles, CA, United States; ^2^Department of Psychiatry and Biobehavioral Sciences, Semel Institute for Neuroscience and Human Behavior, Brain Research Institute, David Geffen School of Medicine, University of California, Los Angeles, Los Angeles, CA, United States; ^3^Edie and Lew Wasserman Center, University of California, Los Angeles, Los Angeles, CA, United States; ^4^Psychology Department, Florida State University, Tallahassee, FL, United States; ^5^Department of Psychology, Social Cognition Center Cologne, University of Cologne, Cologne, Germany

**Keywords:** embodiment, empathy, fMRI, moral dilemmas, moral judgment, process dissociation, neural resonance

## Abstract

The dual process model of moral decision-making suggests that decisions to reject causing harm on moral dilemmas (where causing harm saves lives) reflect concern for others. Recently, some theorists have suggested such decisions actually reflect self-focused concern about causing harm, rather than witnessing others suffering. We examined brain activity while participants witnessed needles pierce another person’s hand, versus similar non-painful stimuli. More than a month later, participants completed moral dilemmas where causing harm either did or did not maximize outcomes. We employed process dissociation to independently assess harm-rejection (deontological) and outcome-maximization (utilitarian) response tendencies. Activity in the posterior inferior frontal cortex (pIFC) while participants witnessed others in pain predicted deontological, but not utilitarian, response tendencies. Previous brain stimulation studies have shown that the pIFC seems crucial for sensorimotor representations of observed harm. Hence, these findings suggest that deontological response tendencies reflect genuine other-oriented concern grounded in sensorimotor representations of harm.

## Introduction

Imagine watching a video of a hypodermic syringe slowly and deliberately piercing a human hand. Despite knowing that the hand belongs to someone else, would you wince and reflexively withdraw your hand, or shrug and stay put? Would you empathize with the person getting pierced? If so, what does your reaction reveal about your moral psychology? The dual-process model of dilemma judgments (Greene et al., 2001) suggests that when people face dilemmas where causing harm saves lives, rejecting such harm (despite not saving lives) reflects other-oriented affective processing. In contrast, judgments to accept harm (thereby maximizing outcomes) reflect cognitive evaluations of outcomes. On such dilemmas, rejecting harm is said to uphold deontological morality, where the morality of actions derives from their intrinsic nature (Kant, 1785/1959), whereas accepting harm is said to uphold utilitarian morality, where the morality of actions derives from their outcomes (Mill, 1861/1998). Although considerable evidence supports the dual process model (e.g., Bartels, 2008; Amit and Greene, 2012; Conway and Gawronski, 2013; Gleichgerrcht and Young, 2013; *cf.* Kahane, 2015), some theorists have questioned whether the affective processing involved in harm-rejection truly reflects other-oriented concern. Affective reactions to harm in dilemma judgments may reflect self-focused emotions centered on causing harm, rather than genuine concern generated by others in pain (Miller et al., 2014).

When people know that others are experiencing pain, sensorimotor and affective systems in their brains respond as though they are personally experiencing pain (Singer et al., 2004; Avenanti et al., 2005; Lamm et al., 2011). This phenomenon, called *neural resonance* (Zaki and Ochsner, 2012), has also been documented for disgust (Wicker et al., 2003; Jabbi et al., 2007), emotions (Carr et al., 2003; Pfeifer et al., 2008), and motor behavior (Fadiga et al., 1995). A key moderator for the presence or absence of activity in sensorimotor circuits seems to be the direct observation of others experiencing pain (Avenanti et al., 2005). When others’ pain is communicated through symbolic cues (e.g., lightning bolts) rather than direct observation, affective circuits are activated, but sensorimotor circuits are not (Singer et al., 2004).

Although researchers conceptualize neural resonance as a component of empathy (Zaki and Ochsner, 2012), they have primarily examined immediate reactions to real-time stimuli, such as smiling faces or video clips of hands getting pierced by needles, rather than personality traits or decision-making tendencies in unrelated contexts. Yet, recent findings suggest that individual differences in neural resonance predict other aspects of an individual’s traits and behavior. Specifically, neural resonance for pain correlates with peoples’ tendency to take others’ perspectives and feel distressed by harm to others (Avenanti et al., 2009). Neural resonance for pain also correlates with charitable donations (Ma et al., 2011), helping behavior (Hein et al., 2011; Morelli et al., 2014), and generosity in economic games where strategic giving does not play any role (Christov-Moore and Iacoboni, 2016). Since correlation is not causation, we have further tested the meaning of these correlations with disruptive brain stimulation, showing that it is possible to modulate generosity by stimulating brain areas whose activity correlates with offers (Christov-Moore et al., 2017). Furthermore, deontological response tendencies on moral dilemmas correlate with empathic concern and perspective-taking (e.g., Conway and Gawronski, 2013; Gleichgerrcht and Young, 2013). These findings suggest that the neural mechanisms associated with pre-reflective reactions to others’ internal states may also be relevant to moral decision-making.

We propose that *people who show greater neural resonance with the pain of others should evince stronger tendencies to reject harm in moral dilemmas*, in line with the dual-process model (Greene et al., 2001). This finding would clarify that internal representations of others’ states (rather than just self-focused emotions) contribute to moral dilemma judgments. Such effects should pertain only to harm-rejection (deontological) response tendencies, which are linked to affective processing, rather than outcome-maximization (utilitarian) response tendencies, which are linked to cognitive processing (Conway and Gawronski, 2013). To examine this possibility, we employed process dissociation (PD, Jacoby, 1991) to disentangle the impacts of harm-rejection and outcome-maximization tendencies on conventional relative dilemma judgments (for a review of PD, see Payne and Bishara, 2009).

**FIGURE 1:**
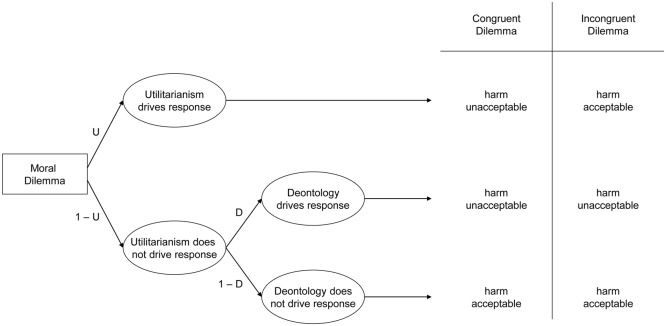
Processing tree illustrating the underlying components leading to judgments that harmful action is either acceptable or unacceptable in congruent and incongruent moral dilemmas, allowing researchers to estimate utilitarian response tendencies (maximize outcomes regardless of whether doing so causes harm or not) and deontological response tendencies (reject causing harm regardless of whether doing maximizes outcomes or not).

In the current work, we recorded participants’ brain activation while they watched videos of needles piercing hands (versus hands gently touched with a Q-tip). A month later or more, participants completed the PD moral dilemma battery, containing 10 moral dilemmas, each with two versions (Conway and Gawronski, 2013). Each dilemma entails causing harm to achieve a particular outcome: incongruent dilemmas correspond to conventional relative moral dilemmas where causing harm maximizes outcomes (hence deontological and utilitarian considerations conflict); congruent dilemmas involve causing harm that does not maximize outcomes (so deontological and utilitarian considerations align – both suggest rejecting harm). Participants indicated whether each harmful action was appropriate or not appropriate. By applying participant responses to both kinds of dilemmas to a processing tree (see **Figure 1**), we can mathematically represent the probability of accepting or rejecting harm in each case, and algebraically combine these equations to solve for two previously unknown variables: deontological inclinations (reflecting a pattern of consistently rejecting harm regardless of whether doing so maximizes outcomes), and utilitarian inclinations (reflecting a pattern of maximizing outcomes, whether or not doing so entails causing harm).

Conway and Gawronski (2013) found that deontological response tendencies uniquely correlated with measures of other-oriented concern, such as empathic concern and perspective-taking (Davis, 1983) and were uniquely increased when viewing photos of the victims of harm, clarifying similar findings on conventional dilemma judgments (e.g., Bartels, 2008; Gleichgerrcht and Young, 2013). Conversely, utilitarian response tendencies uniquely correlated with measures of cognitive processing, such as need for cognition (Epstein et al., 1996), and are uniquely impaired by cognitive load, again clarifying similar findings on relative judgments (e.g., Greene et al., 2008; Moore et al., 2008). Meta-analytic results indicated that deontological and utilitarian response tendencies are typically uncorrelated, but each correlates with conventional relative dilemma judgments in opposite directions (Friesdorf et al., 2015), suggesting that the PD parameters reflect two independent response tendencies that jointly influence conventional relative dilemma judgments.

Given research suggesting that deontological dilemma response tendencies reflect relatively affective other-oriented concern, whereas utilitarian dilemma response tendencies reflect relatively cognitive outcome-focused processing, we predicted that when viewing others in pain, activation in brain regions associated with pain processing would predict deontological, but not utilitarian, response tendencies. However, if Miller et al. (2014) are correct that deontological decisions primarily reflect self-focused concerns over causing harm, rather than other-focused affective processing regarding witnessing harm, then brain activation while witnessing others in pain (pain one did not cause) should not predict dilemma response tendencies.

Moreover, we aimed to clarify the nature of other-oriented concern involved in deontological dilemma decisions. As mentioned above, neural resonance for pain recruits both affective (anterior cingulate, medial prefrontal cortex, and anterior insula) and sensorimotor (ventral premotor cortex, inferior parietal lobe, and primary motor cortex) neural systems to different extents depending on stimuli and context (reviewed in Lamm et al., 2011). We employed stimuli known to activate both sensorimotor and affective systems (Avenanti et al., 2005; Bufalari et al., 2007) – videos of painful stimuli applied to others (Avenanti et al., 2005) – to examine which system best predicts deontological dilemma response tendencies, rather than employing stimuli known to selectively activate affective but not sensorimotor networks (as in Singer et al., 2004), which would prevent us from testing these hypotheses against one another.

If affective systems best predict deontological tendencies, this may suggest that deontological decisions primarily reflect general affective responses to others’ pain. On the other hand, if sensorimotor circuits best predict deontological tendencies, this may suggest that deontological decisions are partially grounded in participants’ previous personal perceptual and motor experiences of pain. In other words, witnessing a needle pierce someone else’s hand may lead participants to imagine, albeit likely implicitly, how others experience the physical sensation of having their hand pierced. Such physiological representations of others’ pain may lead participants to reject actions that cause others pain – such as endorsing deontological moral dilemma judgments. Note that whereas previous functional magnetic resonance imaging (fMRI) dilemma research examined brain activation as participants completed the dilemma task itself (e.g., Greene et al., 2001, 2004), we examined brain activation in response to unrelated stimuli – the needle task – and used this activation to predict dilemma responses made over 1 month later.

To recapitulate, this study tested two hypotheses. First, we examined whether neural responses to witnessing another person experience pain predicted subsequent deontological (but not utilitarian) dilemma response tendencies. Second, we investigated the nature of the functional processes associated with deontological tendencies: whether they are best predicted by systems involved in general affective processing, or by systems involved in the sensorimotor processing of watching others in pain.

## Materials and Methods

### Participants

We recruited 19 ethnically diverse adults aged 18–35 (9 female), through community fliers. We required that participants were right handed, with no prior or concurrent diagnosis of any neurological (e.g., epilepsy, Tourette’s syndrome), psychiatric (e.g., schizophrenia), or developmental disorders (e.g., ADHD, dyslexia), and no history of drug or alcohol abuse. All recruitment and experimental procedures were performed under approval of UCLA’s institutional review board.

### Functional MRI Procedure

#### Pain Video Task

We employed (with permission) the 27 full-color video stimuli from Bufalari et al. (2007). Each video depicted the same human hand being pierced by a hypodermic syringe in varying locations (Pain condition), being touched by a wooden Q-tip in the same locations (Touch condition), or in isolation (Hand condition). The run consisted of 12 trial blocks lasting 26 s each, plus 8 alternating rest blocks that lasted either 5 or 10 s. Each trial block consisted of four videos of a single condition (Pain, Touch, Hand), each 5 s in duration, with an interstimulus interval of 400 ms. Subjects were simply instructed to watch the video clips. They were assured that the hand in the video clip was a human hand and not a model, but they were not instructed to empathize with the model, nor were there any audiovisual cues to indicate pain from the hand’s owner. We used (and controlled for) three different block orders, and ensured an approximately equal proportion of male and female subjects viewed each order. We coded the task within Presentation (created by Neurobehavioral Systems).

#### MR Image Acquisition

The fMRI data were acquired on a Siemens Trio 3 Tesla system housed in the Ahmanson-Lovelace Brain Mapping Center at UCLA. Functional images were collected over 36 axial slices covering the whole cerebral volume using an echo planar T2^∗^-weighted gradient echo sequence (TR = 2500 ms; TE = 25 ms; flip angle = 90°; matrix size = 64 × 64; FOV 20 cm; in-plane resolution = 3 mm × 3 mm; slice thickness = 3 mm/1 mm gap). Additionally, a high-resolution T1-weighted volume was acquired in each subject (TR = 2300 ms; TE = 25 ms; TI = 100 ms; flip angle = 8°; matrix size = 192 × 192; FOV = 256 cm; 160 slices), with approximately 1 mm isometric voxels (1.3 mm × 1.3 × 1.0 mm).

#### Functional MRI Analysis

Analyses were performed in FMRI Expert Analysis Tool (FEAT), part of FMRIB’s Software Library (FSL)^1^. After motion correction using MCFLIRT, images were temporally high-pass filtered with a cutoff period of 70 s (approximately equal to one rest-task-rest-task period), and smoothed using a 6 mm Gaussian FHWM algorithm in three dimensions. Each participants’ functional data were co-registered to standard space (MNI 152 template) via registration of an averaged functional image to the high resolution T1-weighted volume using a six degree-of-freedom linear registration and of the high-resolution T1-weighted volume to the MNI 152 template via non-linear affine registration, implemented in FNIRT.

In order to remove non-neuronal sources of coherent oscillation in the relevant frequency band (0.01–0.1 Hz), preprocessed data were subjected to probabilistic independent component analysis as implemented in Multivariate Exploratory Linear Decomposition into Independent Components (MELODIC) Version 3.10, part of FSL^2^. Noise components corresponding to head motion, scanner noise, and aliasing of cardiac/respiratory signals were identified by observing their localization, time series, and spectral properties (after Kelly et al., 2010) and removed using FSL’s regfilt command.

We performed statistical analyses of fMRI data using FSL’s implementation of the general linear model. For first level analyses gauging task activation, the anticipated BOLD response to each condition was modeled using an explanatory variable (EV) consisting of a boxcar function describing the onset and duration of each relevant experimental condition (task conditions, rest, and instruction screen) convolved with a canonical double-gamma hemodynamic response function (HRF) to produce an expected BOLD response. The temporal derivative of each task EV was also included in the model to improve the model fit and accommodate regional variations in the BOLD signal. Functional data were then fitted to the modeled BOLD signal using FSL’s implementation of the general linear model. This produced parameter estimate maps describing the goodness-of-fit of the data to the modeled BOLD signal. Contrasts were then performed to isolate clusters of voxels that showed significantly different parameter estimates between each condition. Higher-level analyses were implemented by including subjects’ scores on the deontological and utilitarian parameters as separate EV’s against subjects’ parameter estimate maps (superimposed in standard space) for the contrast Pain > Touch. Resultant whole-brain parameter estimates representing the between-subject variance explained by each behavioral EV were converted to normalized *Z*-scores and corrected for multiple comparisons at the cluster level (using Gaussian random field theory) using a cluster-wise *Z*-threshold of 2.3 and *p*-value cutoff of 0.05, using FLAME 1 + 2. The resultant *Z*-statistic images from the higher-level analysis were then masked by the activation map for the contrast Pain > Touch. The rationale for this masking procedure is straightforward: we were testing the hypothesis that task-related activation of neural systems engaged in processing others’ pain contributes to decision-making in moral dilemmas. Task-irrelevant areas cannot speak to this hypothesis.

### Process Dissociation Dilemma Battery

In a separate experimental session conducted at least 1 month following the neuroimaging protocol, participants completed a battery of 10 moral dilemmas, each with two versions: an incongruent and congruent version (Conway and Gawronski, 2013). *Incongruent* moral dilemmas correspond to traditional, high-conflict moral dilemmas commonly employed in research (e.g., Foot, 1967; Koenigs et al., 2007). In such dilemmas, participants read a scenario where a great deal of harm is impending, but participants could avoid this impending harm by accepting causing a lesser degree of harm. For example, in the crying baby dilemma, townspeople are hiding from murderous soldiers, but a baby is about to cry, which will summon the soldiers who will murder the townspeople. The actor could fatally smother the baby to prevent its cries, thereby saving the townspeople. Other dilemmas include torturing a person to prevent a bomb from killing several people, and causing severe harm to research animals to cure AIDS. Participants indicated whether causing each harm in order to achieve the specified outcome is *appropriate* or *not appropriate* (in line with Greene et al., 2001).

*Congruent* dilemmas are worded identically to incongruent dilemmas, except the outcome of causing harm has been minimized. For example, in the congruent version of the crying baby dilemma, the actor could kill the baby to prevent the townspeople from being forced to performing hard labor. In other dilemmas, the actor could torture a person to prevent a messy but non-lethal paint bomb, or cause severe harm to research animals to create a better facial cleanser. Again, participants indicated whether causing harm to achieve the specified outcome in acceptable or not acceptable. Participants responded to all dilemmas in the same fixed random order as Conway and Gawronski (2013). By considering participant responses to both incongruent and congruent dilemmas, it is possible to perform a PD analysis (Jacoby, 1991) that provides independent estimates of each participant’s inclination to reject causing harm (consistent with deontology), that appears to track affective reactions to harm, as well as inclinations to maximize outcomes (consistent with utilitarianism), that appears to track cognitive evaluations of outcomes (Conway and Gawronski, 2013).

We computed the deontological and utilitarian PD parameters using the six formulae described by Conway and Gawronski (2013). Consider **Figure 1**. The top path (*U*) illustrates the case where utilitarianism drives responses: this entails rejecting harm for congruent dilemmas but accepting harm for incongruent dilemmas (thus always maximizing outcomes). Representing the case where utilitarianism drives responses also allows representing the case where utilitarianism does not drive responses: (1-*U*). This case may be further subdivided into (1) the case where deontology drives responses (1-*U* ×*D*), which entails rejecting harmful actions in both congruent and incongruent dilemmas (thus always rejecting causing harm), as well as (2) the case where neither utilitarianism nor deontology drives responses (1-*U* × 1-*D*), which entails accepting harm for both congruent and incongruent dilemmas (suggesting at best an amoral insensitivity to the outcomes of one’s actions, or at worst general willingness to cause harm even when doing so makes the world worse overall).

Using the processing tree in **Figure 1**, researchers can algebraically represent each case: when participants accept or reject harm on congruent or incongruent dilemmas. For congruent dilemmas, participants may reject harm either when utilitarianism drives responses (*U*), or when utilitarianism does not, but deontology does (1-*U* ×*D*). Conversely, participants may accept harm only when neither utilitarianism nor deontology drives responses (1-*U* × 1-*D*). For incongruent dilemmas, participants reject harm when utilitarianism does not drive the response, but deontology does (1-*U × D*). Conversely, people may accept harm either when utilitarianism drives responses (*U*), or when neither utilitarianism nor deontology drives responses (1-*U* × 1-*D*). By combining these values, researchers can algebraically represent the cases when participants accept and reject harm on congruent or incongruent dilemmas. The probability of rejecting harm for congruent dilemmas is represented by the case where either utilitarianism drives the response, or when utilitarianism does not drive the response, but deontology does:

(1) p(unacceptable|congruent)=U+[(1−U)×D]

Conversely, the probability of accepting harm for congruent dilemmas is represented by the case that neither utilitarianism nor deontology drives the response:

(2) p(acceptable|congruent)=(1−U)×(1−D)

For incongruent dilemmas, the probability of rejecting harm is represented by the case that deontology drives the response when utilitarianism does not:

(3) p(unacceptable|incongruent)=(1−U)×D

Conversely, the probability of accepting harm for incongruent dilemmas is represented by the cases that utilitarianism drives the response, and neither deontology nor utilitarianism drives the response:

(4) p(acceptable|incongruent)=U+[(1−U)×(1−D)]

Next, researchers can enter the empirical distributions of participant’s *acceptable* and *unacceptable* responses to congruent and incongruent dilemmas into these equations, and then algebraically combine them to solve for the two parameters. By including Eq. 3 into Eq. 1, researchers can solve for *U*:

(5) U=p(unacceptable|congruent)−p(unacceptable|incongruent)

Finally, by including the value for *U* in Eq. 3, researchers can solve for *D*:

(6) D=p(unacceptable|incongruent)/(1−U)

Together, these formulas enable researchers to independently estimate the degree to which participants systematically rejected causing harm (deontology parameter) and systematically maximize outcomes (utilitarian parameter).

## Results

Participants’ scores on the moral dilemma battery were process dissociated to produce the Deontological Parameter (*M* = 0.45, *SD* = 0.087), and the Utilitarian Parameter (*M* = -0.41, *SD* = 0.163). Neither scale violated the assumption of normality as verified via a Shapiro–Wilk’s test (**Supplementary Figure S1**).

**FIGURE 2:**
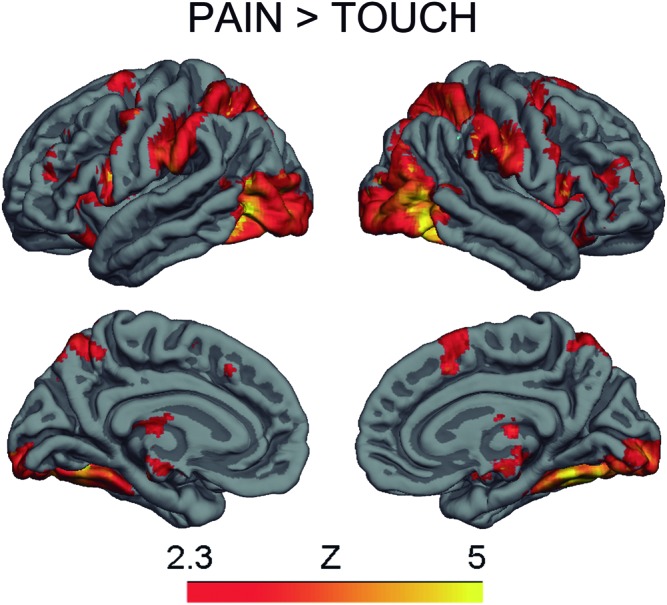
BOLD activation for the contrast Pain > Touch. Heat maps reflect *Z*-scores.

**FIGURE 3:**
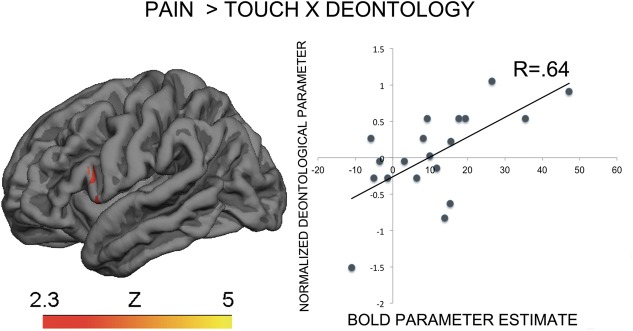
BOLD activation in the inferior premotor cortex for the contrast Pain > Touch predicts the deontological tendency in moral dilemmas. Heat map reflects *Z*-scores of normalized goodness-of-fit of normalized deontological process dissociation (PD) parameter to parameter estimates of BOLD activation for the contrast of interest. The descriptive scatterplot presents the relationship between the average BOLD parameter estimate in the premotor clusters and the normalized deontological PD parameter. The *R*-value reflects a Pearson’s correlation between the average beta-parameter estimate in the premotor clusters and the deontological parameter and is purely illustrative.

The contrast Pain > Touch yielded clusters of activation consistent with prior studies on empathy for pain (Lamm et al., 2011). These clusters were located in visual cortex, inferior and superior parietal cortex, motor cortex, inferior premotor cortex, dorsolateral prefrontal cortex, anterior cingulate, thalamus, and others (see **Figure 2** and **Supplementary Table S1**). The deontological PD parameter (reflecting harm-aversion dilemma judgments) correlated with BOLD signal changes in the left inferior frontal gyrus, pars opercularis, (see **Figure 3** and **Table 1**). As predicted, there were no correlations between the utilitarian PD parameter (reflecting outcome-maximization judgments) and BOLD signal changes for the contrast Pain > Touch.

**Table 1:** Clusters of high goodness-of-fit when regressing the deontological parameter against parameter estimates for Pain > Touch across subjects.

Brain region	*x*	*y*	*z*	*Z*	Voxels
Left inferior frontal gyrus, pars opercularis	-58	16	14	3.65	173
Left inferior frontal gyrus, pars opercularis	-64	8	-2	3.77	145
Left inferior frontal gyrus, pars opercularis	-52	12	2	3.21	18

*Coordinates are in MNI_152 space, reported in millimeters; normalized goodness-of-fit is reported with *Z*-scores.*

## Discussion

This study is the first attempt to assess the relationship between the BOLD signal while people witness others in pain and those same peoples’ later response tendencies in hypothetical moral dilemmas. It belongs to a larger project on the relationships between type 1, pre-reflective responses to the internal states of other people, and type 2, reflective prosocial decisions that also includes two recent studies (Christov-Moore and Iacoboni, 2016; Christov-Moore et al., 2017). A broad array of brain regions responded to the pain video task, but of those, later harm-rejection (deontological) judgment tendencies were only predicted by activation in a sensorimotor area (IFG) associated with observing, imitating, and understanding the motor behavior of others (Iacoboni, 2009; Urgesi et al., 2014), their internal states (Carr et al., 2003; Lamm et al., 2011), and the broader construct of empathy (Carr et al., 2003; Lamm et al., 2011) and empathic accuracy (Paracampo et al., 2017). In contrast, areas associated with affective processing of empathy for pain, such as the anterior cingulate or anterior insula (Singer et al., 2004), did *not* predict deontological responses, despite those areas showing activation during the pain video task. As expected, no brain activation while witnessing pain correlated with outcome-maximization (utilitarian) judgment tendencies. These findings cannot reflect priming effects from the pain video task, as participants completed the dilemma battery at least 1 month following the scanner session. Together, results suggest that people who demonstrate greater sensorimotor empathy for observed pain in others are more likely to avoid causing harm (but not maximize outcomes) in hypothetical moral dilemmas.

These results, while correlational, support the hypothesis that deontological response tendencies primarily reflect genuine concern for others generated by witnessing pain, rather than self-focused emotional reactions to *causing* pain – after all, participants passively witnessed, rather than actively caused, the painful needle stimuli (Miller et al., 2014). These findings align with research linking deontological response tendencies to other-oriented processing, such as empathic concern and perspective-taking (e.g., Bartels, 2008; Conway and Gawronski, 2013; Gleichgerrcht and Young, 2013), thereby corroborating the dual-process model (Greene et al., 2001). However, these findings do not rule out the possibility that self-focused concerns regarding causing harm independently influence deontological response tendencies via mechanisms not captured in the current paradigm (Reynolds and Conway, in press).

Furthermore, these findings suggest the possible nature of the functional processes supporting other-oriented affective responses that lead to deontological choices. We employed stimuli known to elicit activation in circuits involved in both sensorimotor and general affective processing, and indeed found activation in both regions during the pain video task. However, only activation in the posterior inferior frontal cortex (pIFC) – associated with sensorimotor processing but not general affective processing – predicted subsequent deontological response tendencies. Past work shows that the pain video used here modulated transcranial magnetic stimulation (TMS)-induced motor evoked potentials (MEPs), an index of motor excitability, providing clear evidence for a sensorimotor component to empathy (Avenanti et al., 2005). TMS-induced MEP modulation while perceiving others’ hand actions, however, disappears when pIFC and neighboring areas are transiently disrupted by repetitive TMS (Avenanti et al., 2007). These findings suggest that activity in pIFC and neighboring areas is crucial for the sensorimotor component of empathy (Avenanti et al., 2005). Hence, we believe the correlation between deontological tendencies and pIFC activity in the current study is mainly driven by the pIFC’s role in the sensorimotor component of empathy. Furthermore, the role of the IFG in recognizing and understanding the behavior of others (Urgesi et al., 2014) as well as accurately assessing their internal states or empathic accuracy (Paracampo et al., 2017) is consistent with the notion that the pIFC integrates or provides links between high- and low-level empathic processes like neural resonance and moral decision-making.

We propose that the sensorimotor and affective mechanisms used to process one’s own pain also react to abstract and non-sensory harm to others, such as vivid images of pain – including mental images conjured when considering hypothetical moral dilemma scenarios. The more individuals recruit non-agent-specific pain representations in response to others’ suffering, the more likely they are to personally appreciate how others will experience harm. People who especially engage in such implicit representations appear especially likely to recoil from inducing pain on subsequent moral dilemma tasks (i.e., reject causing even harm that maximizes outcomes). Such findings are consistent with evidence that making harm vivid reduces willingness to directly inflict harm in moral dilemmas (e.g., Bartels, 2008), specifically through increased deontological inclinations (Conway and Gawronski, 2013), and that impairing visual processing reduces deontological judgments (Amit and Greene, 2012). However, the present findings are the first to suggest that the embodied consideration of other people’s pain at a sensorimotor level influence moral decision-making.

Obviously, these findings do not challenge the engagement of affective systems in moral dilemma judgment tendencies. Indeed, we should exercise caution extrapolating from the current limited sample. The lack of brain-behavior correlation with affective systems may reflect a false negative due to insufficient power, or else indicate that perhaps affective processing in moral dilemmas is relatively insensitive to individual differences in judgment tendencies. Furthermore, because no subjective evaluation was conducted regarding subject’s internal state while observing the needle stimuli, we cannot be sure that the task we used consistently tapped into concern for others, rather than self-focused emotional/sensorimotor experience. The relationship between these sensorimotor representations and dilemma responses may not be the one hypothesized here. However, the vicarious nature of this sensorimotor processing as well as past studies showing correlations between neural activity during similar tasks and other-oriented processes like empathy and pro-sociality mitigate this concern.

As predicted, these results failed to indicate an association between brain activation while witnessing harm and utilitarian response inclinations. This pattern meshes with previous findings suggesting the utilitarian responses primarily reflect abstract cognitive considerations of overall well-being, rather than a concrete focus on harm (e.g., Greene et al., 2008; Moore et al., 2008; Conway and Gawronski, 2013; *cf.* Kahane, 2015). Future research might profitably examine whether the utilitarian PD parameter correlates with brain activity when participants engage in theory of mind reasoning, mathematical calculations, or other abstract cognitive operations.

The current findings bolster the growing evidence that individual variability in sensorimotor and affective neural resonance predict differences in decision-making (Hein et al., 2011; Ma et al., 2011; Morelli et al., 2014; Christov-Moore and Iacoboni, 2016). Thus far, researchers have investigated this link only in the realm of prosocial decision-making (decisions involving potential gains); the current findings extend this relationship to moral dilemma judgments (decisions involving potential losses). Whereas some studies have implicated affective areas like the anterior insula in motivating prosocial decisions (Hein et al., 2011), others have implicated sensorimotor areas like the superior parietal lobe (Christov-Moore and Iacoboni, 2016) and SII/pIFC (Ma et al., 2011). One interpretation of this pattern suggests that general affective processing may be more important for motivating prosocial behavior rather than harm avoidance, whereas sensorimotor processing features for both. Alternatively, it remains possible that the current stimuli do not reveal the full extent to which general affective processing impacts harm-avoidance judgments. Consider that the videos we employed showed a close-up of hands getting pierced, without accompanying stimuli associated with witnessing others in pain (e.g., facial grimacing, cries of pain). Perhaps including such cues may provide increased evidence for the impact of general affective processing on dilemma decision-making. It remains likely that the relative contributions of affective or sensorimotor processes depend on multiple factors.

## Conclusion

These findings are the first to demonstrate that, upon witnessing others in pain, brain activation in sensorimotor circuits involved in processing pain in the self predicts subsequent inclinations to avoid causing harm in moral dilemmas (even to maximize outcomes). Such findings clarify the importance of genuine concern for others in dilemma judgments, corroborate the dual process model, and provide further evidence for the utility of PD in dilemma decision-making. Moreover, these findings suggest a role for the embodied sensorimotor processing of harm when making moral dilemma decisions. People with stronger internal sensorimotor representations of others’ physical pain may be especially unwilling to visit harm on others, even for a good cause.

## Author Contributions

LC-M developed the study concept. All authors contributed to the study design. Testing and data collection were performed by LC-M and PC. LC-M and PC performed the data analysis and interpretation under the supervision of MI. LC-M and PC drafted the manuscript, and MI provided critical revisions. All authors approved the final version of the manuscript for submission.

## Conflict of Interest Statement

The authors declare that the research was conducted in the absence of any commercial or financial relationships that could be construed as a potential conflict of interest.

## Footnotes

1 www.fmrib.ox.ac.uk/fsl

## Supplementary Material

FIGURE S1 Distributions of deontological and utilitarian moral dilemma response tendencies. Solid line indicates ideal normal distribution. Mean and standard deviation are presented for both response tendency subscales.

TABLE S1 MNI coordinates (in mm) of representative peaks of activation for the contrast Pain > Touch.
