# Supplementary figures and images for: Deontological Dilemma Response Tendencies and Sensorimotor Representations of Harm to Others
Source: Front Integr Neurosci. 2017 Dec 12;11:34. doi: 10.3389/fnint.2017.00034 (PMC5733021; doi:10.3389/fnint.2017.00034)

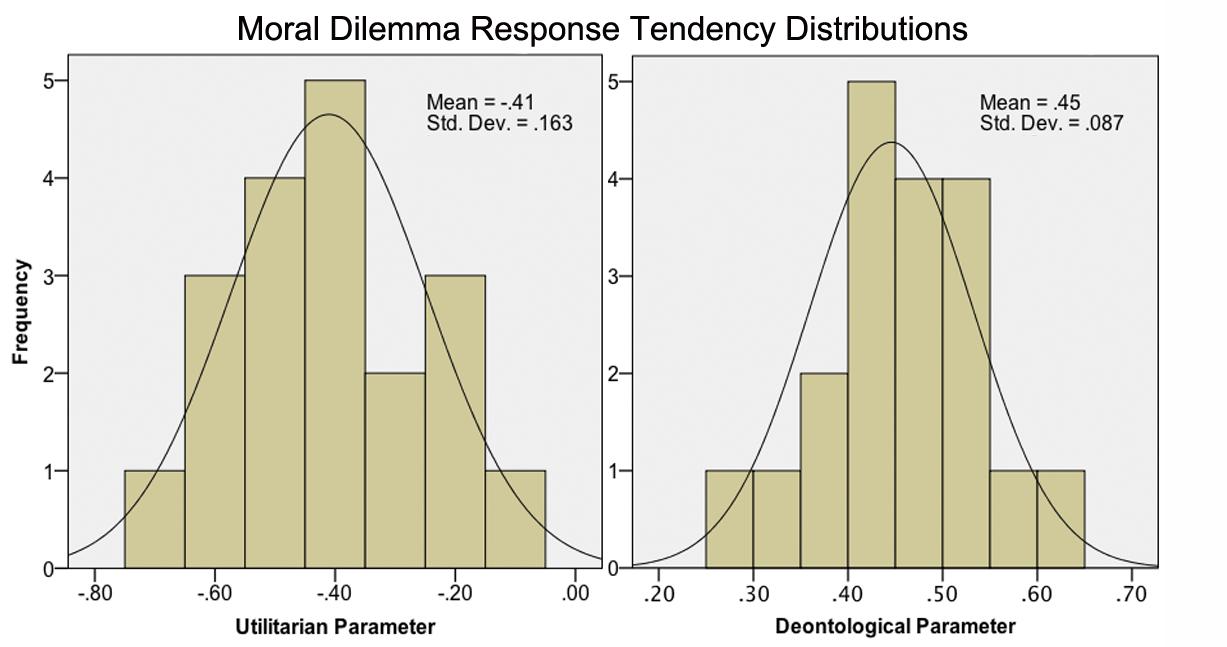

Supplement: FIGURE S1 — Distributions of deontological and utilitarian moral dilemma response tendencies. Solid line indicates ideal normal distribution. Mean and standard deviation are presented for both response tendency subscales. [file Image_1.TIF]
