# Supplementary material for: Deontological Dilemma Response Tendencies and Sensorimotor Representations of Harm to Others
Source: Front Integr Neurosci. 2017 Dec 12;11:34. doi: 10.3389/fnint.2017.00034 (PMC5733021; doi:10.3389/fnint.2017.00034)
Supplement: TABLE S1 — MNI coordinates (in mm) of representative peaks of activation for the contrast Pain > Touch. [file Table_1.docx]

**Supplementary Table 1**

MNI coordinates (in mm) of representative peaks of activation for the contrast Pain>Touch

| **Brain Region** | **x** | **y** | **z** | **Z** |
| --- | --- | --- | --- | --- |
| L Lateral Occipital Cortex | -44 | -68 | -6 | 7.33 |
| R Fusiform Gyrus | 32 | -68 | -18 | 7.12 |
| R Inferior Temporal Gyrus | 44 | -56 | -10 | 7.02 |
| R Temporal Occipital Fusiform Cortex | 44 | -52 | -12 | 6.29 |
| L Temporal Occipital Fusiform Cortex | -34 | -62 | -14 | 5.94 |
| R Supramarginal Gyrus | 62 | -28 | 32 | 5.77 |
| R Lateral Occipital Cortex | 32 | -80 | 28 | 5.65 |
| L Fusiform Gyrus | -32 | -64 | -14 | 5.62 |
| L Inferior Frontal Gyrus, pars opercularis | -50 | 8 | 16 | 5.07 |
| R Lateral Occipital Cortex | 52 | -64 | 2 | 4.89 |
| R Precentral Gyrus | 52 | 2 | 38 | 4.82 |
| Paracingulate Gyrus | 4 | 18 | 46 | 4.73 |
| L Precentral Gyrus | -50 | 6 | 26 | 4.69 |
| L Superior Parietal Lobe | -34 | -46 | 56 | 4.52 |
| L Supramarginal Gyrus | -66 | -24 | 26 | 4.41 |
| R Frontal Operculum | 36 | 20 | 8 | 4.26 |
| L Occipital Pole | -10 | -98 | -8 | 4.12 |
| R Inferior Frontal Gyrus, pars opercularis | 52 | 12 | 8 | 4.11 |
| R Occipital Pole | 12 | -94 | -4 | 3.96 |
| R Putamen | 22 | 4 | 0 | 3.93 |
| L Anterior Insula | -30 | 20 | 6 | 3.91 |
| R Postcentral Gyrus | 54 | -16 | 38 | 3.91 |
| R Superior Parietal Lobe | 36 | -40 | 52 | 3.88 |
| R Posterior Insula | 44 | 2 | 2 | 3.76 |
| R Pallidum | 20 | 4 | 0 | 3.68 |
| R Anterior Insula | 38 | 14 | 2 | 3.67 |
| L Pallidum | -18 | -2 | 0 | 3.62 |
| L Postcentral Gyrus | -60 | -26 | 40 | 3.61 |
| L Frontal Operculum | -32 | 22 | 10 | 3.57 |
| L Posterior Insula | -42 | -4 | 0 | 3.54 |
| L Thalamus | -6 | -18 | 0 | 3.5 |
| Anterior Cingulate | 2 | 16 | 42 | 3.24 |
| R Orbitofrontal Cortex | 30 | 24 | -20 | 3.22 |
| R Thalamus | 6 | -22 | 0 | 2.89 |
| L Orbitofrontal Cortex | -28 | 34 | -20 | 2.47 |
